# Angiotensin Converting Enzyme Regulates Cell Proliferation and Migration

**DOI:** 10.1371/journal.pone.0165371

**Published:** 2016-12-19

**Authors:** Erika Costa de Alvarenga, Matheus de Castro Fonseca, Clarissa Coelho Carvalho, Rodrigo Machado Florentino, Andressa França, Eveline Matias, Paola Bianchi Guimarães, Carolina Batista, Valder Freire, Adriana Karaoglanovic Carmona, João Bosco Pesquero, Ana Maria de Paula, Giselle Foureaux, Maria de Fatima Leite

**Affiliations:** 1 Department of Physiology and Biophysics, Federal University of Minas Gerais, Belo Horizonte, MG, Brazil; 2 Department of Natural Sciences, Federal University of São João del Rei, São João Del Rey, MG, Brazil; 3 Department of Physics, Federal University of Ceará, Fortaleza, CE, Brazil; 4 Department of Biophysics, Federal University of São Paulo, São Paulo, SP, Brazil; 5 Department of Physics, Federal University of Minas Gerais, Belo Horizonte, MG, Brazil; 6 Department of Morphology, Federal University of Minas Gerais, Belo Horizonte, MG, Brazil; Texas Technical University Health Sciences Center, UNITED STATES

## Abstract

**Background:**

The angiotensin-I converting enzyme (ACE) plays a central role in the renin-angiotensin system, acting by converting the hormone angiotensin-I to the active peptide angiotensin-II (Ang-II). More recently, ACE was shown to act as a receptor for Ang-II, and its expression level was demonstrated to be higher in melanoma cells compared to their normal counterparts. However, the function that ACE plays as an Ang-II receptor in melanoma cells has not been defined yet.

**Aim:**

Therefore, our aim was to examine the role of ACE in tumor cell proliferation and migration.

**Results:**

We found that upon binding to ACE, Ang-II internalizes with a faster onset compared to the binding of Ang-II to its classical AT1 receptor. We also found that the complex Ang-II/ACE translocates to the nucleus, through a clathrin-mediated process, triggering a transient nuclear Ca^2+^ signal. *In silico* studies revealed a possible interaction site between ACE and phospholipase C (PLC), and experimental results in CHO cells, demonstrated that the β3 isoform of PLC is the one involved in the Ca^2+^ signals induced by Ang-II/ACE interaction. Further studies in melanoma cells (TM-5) showed that Ang-II induced cell proliferation through ACE activation, an event that could be inhibited either by ACE inhibitor (Lisinopril) or by the silencing of ACE. In addition, we found that stimulation of ACE by Ang-II caused the melanoma cells to migrate, at least in part due to decreased vinculin expression, a focal adhesion structural protein.

**Conclusion:**

ACE activation regulates melanoma cell proliferation and migration.

## Introduction

The renin-angiotensin system (RAS), a peptidergic hormone system, is well known for its role in the regulation of blood pressure, electrolyte balance and vascular remodeling [1, 2, 3]. Renin cleaves angiotensinogen to produce the decapeptide angiotensin (Ang) I. Subsequently, after cleavage of two carboxy-terminal amino acids by the angiotensin I–converting enzyme (ACE), Ang-I is converted into the octapeptide Ang-II. Two distinct forms of ACE are expressed in humans: a somatic form that is abundant on the surface of lung endothelial cells and a smaller isoenzyme found exclusively in testis [4]. The activity of somatic ACE has a crucial role in catalyzing the conversion of Ang-I to the Ang-II, which modulates blood pressure, vasoconstriction, inflammation, cell proliferation and vascular rearrangement [5].

In addition to the classic participation of ACE in the above-mentioned functions, new roles for ACE have recently been described [4, 6, 7]. The canonical Ang-II pathway is mediated by activation of either AT_1_ or AT_2_ receptors, which typically mediate opposite functions [8]. However, recent findings have revealed that, despite the traditional enzymatic functions, ACE is also capable of mediating intracellular signaling. Kohlstedt and colleagues [4] showed that binding of an ACE inhibitor to ACE elicits outside-in signaling in endothelial cells, enhancing the activity of ACE-associated kinase CK2 and increasing the phosphorylation of the intracellular tail of ACE. This in turn promotes the activation of JNK as well as the accumulation of phosphorylated c-Jun in the endothelial cell nucleus that ultimately increases ACE expression *in vitro* and *in vivo*. Such mechanism suggested that ACE activation might control expression of diverse proteins besides ACE itself. Indeed, Kohlstedt *et al*. [6] found that binding of ramipril (ACE inhibitor) to ACE directly induces a signaling cascade that results in the activation of the transcription factor AP-1 and an increase in the expression/activity of cyclooxygenase-2 in endothelial cells. Furthermore, Guimarães *et al*. [7] demonstrated that ACE behaves as a receptor for Ang-II triggering Ca^2+^ signaling, through inositol 1,4,5-trisphosphate (InsP_3_) formation. Accordingly, a binding site for Ang-II was described on ACE [9]. However, the role of Ang-II-mediated signaling through ACE is still unclear. In the current study, we investigated novel roles of ACE as Ang-II receptor, demonstrating that ACE regulates cell proliferation and migration in melanoma cells.

## Materials and Methods

### Material and Reagents

Dulbeccos’s Modified Eagle’s Medium (DMEM), RPMI 1640 medium, penicillin, streptomycin, amphotericin and fetal bovine serum (FBS) were purchased from Gibco (Grand Island, USA). Fluo-4/AM, DAPI, secondary antibodies conjugated to Alexa-488, Alexa-633, Angiotensin-II FITC conjugate and Lipofectamine® 2000 were purchased from Life Technologies (New York, USA); rabbit IgG secondary antibody was purchased from Sigma-Aldrich, (St. Louis, USA). Polyclonal anti-GAPDH and anti-PLC antibodies were purchased from Santa Cruz Biotechnology (Santa Cruz, USA). Monoclonal anti-ACE antibody and mouse anti-Clathrin was obtained from Merck Millipore (Darmstadt, Germany). Anti-BrDU-POD kit was obtained from Calbiochem (Damstadt, Germany). Ambion Silencer kits were purchased from Life Technologies (New, York, USA). Hydromount was purchased from National Diagnostics (St. Louis, USA). Moloney murine leukemia virus was obtained from Invitrogen (California, USA), TaqMan Universal PCR Master Mix from Applied Biosystems (California, USA), enhanced chemiluminescence (ECL-plus Western Blotting Detection System) and peroxidase-conjugated antibodies were purchased from Amersham Biosciences (Buckinghamshire, UK). Hydroxyurea was purchased from Sigma-Aldrich, (St. Louis, USA), enzyme linked immunosorbent assay from Roche Applied Science (Indianapolis, IN). Angiotensin II was purchased from Sigma-Aldrich (St. Louis, USA) and Angiotensin-II FITC was purchased from Thermo Fisher Scientific (Massachusetts, USA).

### Cell Culture

Chinese Hamster Ovary (CHO, kindly supplied by Dr François Alhenc-Gelas from the Institut National de la Santé et de la Recherche Médicale, Paris, France) cells were stably transfected with a plasmid containing the sequence of the human ACE (CHO-ACE), as previously reported [7]. Similarly, CHO-AT_1_ cells were stably transfected with the plasmid pcDNA3, containing the sequence of the human AT_1_. Also, we used Melan-a (murine melanocytes) and TM-5 cells (murine melanoma cells). Cells were cultured in DMEM supplemented with 10% FBS, while melan-a and TM-5 were cultured in RPMI 1640 medium supplemented with 5% FBS. Cells were incubated at 37°C in a humidified atmosphere of 95% air and 5% CO_2_. Medium was changed every 3 or 4 days, and cells were subcultured, between days 6 and 8, by harvesting with trypsin-EDTA. Semi-confluent (80% to 90%) cells were used in all of the studies.

### Preparation of siRNA

Potential target sites within the ACE gene were selected and then searched with NCBI Blast to confirm specificity for the protein. The siRNAs for ACE and scrambled sequence were prepared by a transcriptional-based method using the Ambion Silencer kit (Life Technologies, New York, USA), according to the manufacturer’s instructions. The sense and antisense oligonucleotides of siRNA were, respectively: siRNA ACE 5’ GCA GTA CAA CTC TCT GCT A 3’ and 5’ GCG GAT CAT AAA GAA GCT T 3’; siRNA scramble 5’ GCG ATG AGT AGC ATC TCT A 3’ and 5’GCA TGC GAC GAT GAC ATA A 3’. Validated siRNAs for clathrin heavy chain were obtained from Ambion (Life Technologies, New York, USA). The sense and antisense sequences were, respectively: siRNA cla 5′ UAA UCC AAU UCG AAG ACC AAU 3′ and 5′ GUA UGA UGC UGC UAA ACU A 3′. Single wall carbon nanotubes (CNT) were used to deliver each siRNA as previously described [10, 11]. Cells were used 48 hours after siRNA treatment as indicated.

### Western Blotting

CHO-ACE and TM-5 cells were harvested as described and protein content was quantified according to Bradford protein assay. For GAPDH detection, mouse monoclonal anti-GAPDH antibody was used at a dilution of 1:5000. For ACE detection, a mouse monoclonal antibody was used at dilution of 1:1000. For Clathrin detection, a rabbit monoclonal anti-Clathrin was used at a dilution of 1:1000. The antibody incubation proceeded for 2 hours at room temperature. After washing, blots were incubated with HRP conjugated goat anti-mouse or anti-rabbit IgG secondary antibody at a dilution of 1:5000 at room temperature for 1 hour. Immuno detection was carried out using enhanced chemiluminescence [12].

### Immunofluorescence

Confocal immunofluorescence was performed as described [12]. Briefly, CHO-ACE cells were seeded onto 6 well culture dishes and 24 hours later, treated with 1 μM Ang-II for the indicated times. Cells were fixed with 4% paraformaldehyde, permeabilized with PBS 1X/Triton 0.5% and blocked (PBS, BSA 10%, Triton 0.5%, goat serum 5%) for 1 hour. Cells were then incubated with anti-ACE (1:100), for 2 hours at room temperature. This was followed by incubation with DAPI and the specific secondary antibodies conjugated with Alexa-Fluor 488 or 633 (1:500) for 1 hour. Images were obtained using a Zeiss LSM 510 confocal microscope with 63X, 1.4 NA objective lens (Thornwood, USA) [10, 12].

### Detection of calcium signals

Ca^2+^ signals were evaluated either by line-scanning or time-lapse confocal microscopy as described [13, 14]. Cells were incubated with 6 μM Fluo-4/AM for 20 min at 37°C. Coverslips containing the cells were transferred to a custom-built perfusion chamber and were observed using a 63X, 1.4 NA objective lens under a Zeiss LSM 510 confocal microscope. Fluo-4/AM was excited at 488 nm using a krypton/argon laser. Global Ca^2+^ transients were measured in both the nucleus and the cytosol.

### Internalization assay

Internalization kinetic assays were performed as described previously (modified from Thomas *et al*. [15]). Briefly, the transfected cells were exposed to 0.4 nM of ^3^H-AngII in a receptor-binding buffer for 3 hours at 4°C. Thereafter, cells were extensively washed with ice-cold receptor-binding buffer and placed at 37°C for 2, 5, 10, 20 and 40 min. Incubations were stopped by placing the cells on ice. Acid-released and acid-resistant radioactive were separated and measured [16]. The percentage of internalized ligand at each time point was calculated from the ratio of the acid-resistant specific binding to the total (acid-resistant + acid-released) specific binding.

### Measurement of BrDU incorporation

Proliferation was measured by BrdU incorporation using an enzyme-linked immunosorbent assay (Roche Applied Science, Indianapolis, IN), according to the manufacturer’s instructions.

### Migration assays

Migration experiments were performed as previously described [17, 18]. TM-5 cells were grown in 12-well plates and cultured in serum-free medium for 24 hours before the experiments. The wound was achieved by scratching a pipette tip across the cell monolayer (approximately 1.3 mm in width). Hydroxyurea (1 mM) was always included in the tissue culture media to prevent cell proliferation. Cells were stimulated with 1 μM of Ang-II for the indicated times. The wound area was measured using the Northern Eclipse (Empix, Mississauga, Canada) software, and the percentage of wound closure at each time point was derived by the formula: (1 –[current wound size/initial wound size]) × 100.

### Quantitative RT-PCR analysis of ACE mRNA in TM-5 and melan-a cells

Total RNA (750 ng) was reverse transcribed to cDNA using Moloney murine leukemia virus (Invitrogen, California, USA) according to the manufacturer’s instructions. The reaction product was amplified by real-time PCR on the 7000 Sequence Detection System (ABI Prism, Applied Biosystems, California, USA) using the TaqMan Universal PCR Master Mix (Applied Biosystems, California, USA). The thermal cycling conditions consisted by an initial denaturation step of 95°C for 10 minutes, 50 cycles at 95°C for 15 seconds, and 60°C for 1 minute. The experiments were performed in triplicate for each data point. The ACE mRNA abundance was quantified as a relative value compared to an internal reference - β-actin. The primers used for real-time PCR were: ACE (forward primer: 5’ TGA GAA AAG CAC GGA GGT ATC C 3’; reverse primer: 5’ AGA GTT TTG AAA GTT GCT CAC ATC A 3’); murine β-actin (GenBank accession No. NM007393), forward primer 5’ CTG GCC TCA CTG TCC ACC TT 3’ and reverse primer 5’ CGG ACT CAT CGT ACT CCT GCT T 3’. The ACE and β-actin mRNA expressions were obtained from the cycle threshold (Ct) associated with the exponential growth of the PCR products. Quantitative values for ACE mRNA expression were obtained by the parameter 2^ΔCt^, in which ΔCt represents the subtraction of the β-actin Ct values from the ACE receptor values.

### Measurement of ACE activity using the fluorescent peptide Abz-FRK(Dnp)P-OH

ACE activity measurements in all the transfected and non-transfected cells were performed using the FRET peptide Abz-FRK(Dnp)P-OH as described [19].

### *In silico* studies

To perform the protein-protein docking calculations, we used the structural coordinates of ACE (PDB entry 1O8A) [20] and PLCβ3 (PDB entry 3OHM) [21], obtained by X-ray crystallography. To predict the structures of the complex ACE-PLCβ3 [21] we performed a rigid-body protein-protein docking with the program ZDOCK 3.02, a freely protein docking server. The result presenting the lowest interaction energy was selected and subsequently evaluated as a function of the centroid-centroid distance in order to estimate the potential energy profile for the ACE-PLC β3 complex within the Universal classical force field [22] available in the Forcite Module of Materials Studio 5.5.

### Statistical analysis

The results are expressed as mean values ± SEM, except where otherwise noted. Prism (GraphPad Prism Software, San Diego, CA) and Image J (NIH; Bethesda, MD) softwares were used for data and image analysis, respectively. Statistical significance was tested using One-way ANOVA followed by Bonferroni test, and p value < 0.05 was taken to indicate statistical significance.

## Results

### Binding of Ang-II to ACE leads to the complex internalization

**Fig 1:**
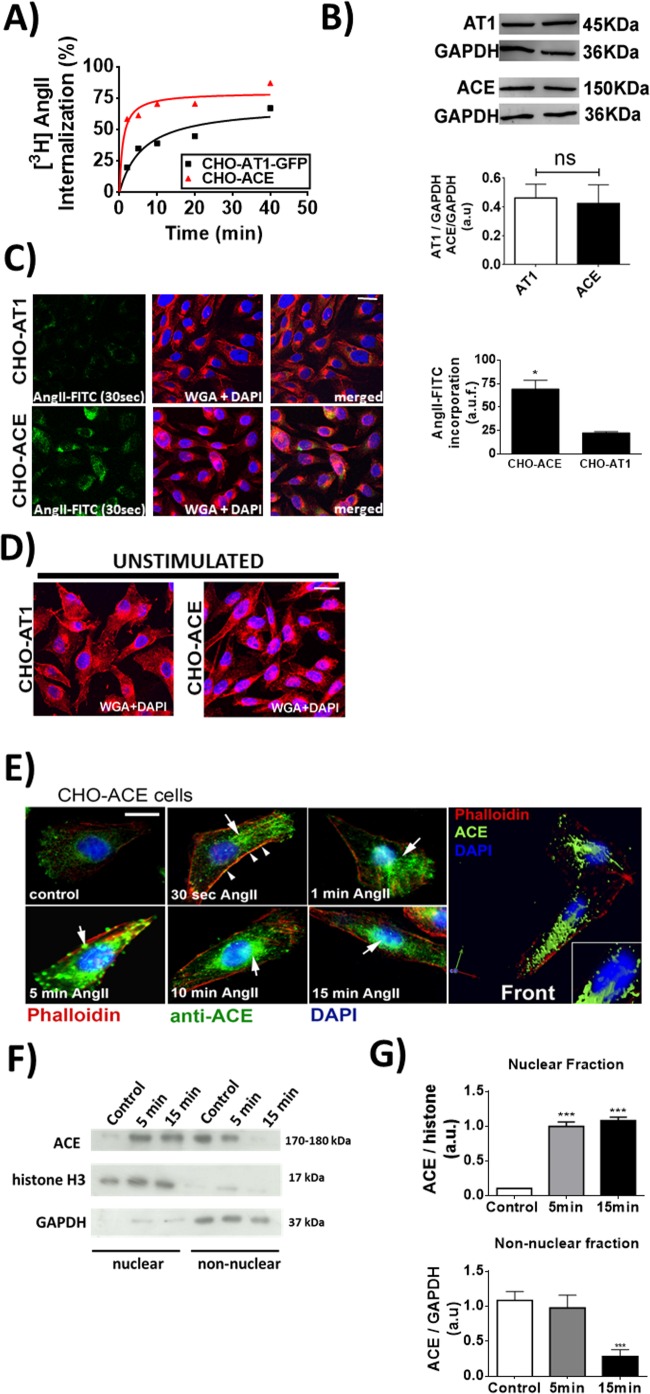
Ang-II induces ACE translocation to the nucleus. (A) Internalization of AT_1_ and ACE in the presence of 4 nM ^3^H-Ang-II. Data are shown as mean from three independent experiments, each performed in duplicate. (B) CHO-ACE and CHO-AT_1_ cells present the same relative protein level of each respective receptor. (Values are mean ± S.E.M, *p<0.05, n = 63 individual experiments). (C) Representative confocal images of internalized Ang-II-FITC (1 μM) in CHO-ACE and CHO-AT_1_ cells after 30 seconds of Ang-II stimulation. DAPI (blue) and Wheat Germ Agglutinin (red), scale bar = 10 μm. On the right, quantification of internalized Ang-II-FITC is presented. Values are mean ± S.E.M, *p<0.05, n = 6. (D) Representative confocal images of unstimulated CHO-ACE and CHO-AT1 cells, labeled for DAPI and WGA. (E) Immunolocalization of ACE after stimulation with Ang-II (1μM), for the indicated times. ACE is shown in green, actin filaments in red, and nucleus in blue (DAPI). Right panel represents a 3D reconstruction of CHO-ACE cell after 15 minutes of incubation with Ang-II (1μM). Scale bar = 10μm. (F) Western blotting of nuclear and non-nuclear protein fractions from CHO-ACE cells, before (control) and after Ang-II (1 μM) stimulation for the indicated times. Histone-3 and GAPDH were used to shown the purification of nuclear and non-nuclear protein fractions, respectively. (G) Densitometry analysis of the western blot. Values are mean ± S.E.M., n = 3 (*** p<0.01).

In addition to the well-known effects of ACE as an enzyme that converts Ang-I to Ang-II [23], ACE has also been reported as a new receptor for Ang-II [7, 9]. In order to investigate the role Ang-II plays through ACE binding, we used CHO cells stably expressing somatic human ACE expression (CHO-ACE cell), in comparison with cells transfected with AT_1_ receptor (CHO-AT_1_ cell). Since it is well known that upon stimulation, AT_1_ receptors internalize [24, 25], we tested whether ACE undergoes a similar process upon Ang-II stimulation. In CHO-AT_1_ and CHO-ACE cells treated with radiolabeled Ang-II (^3^H-Ang-II), we found a more pronounced ^3^H-Ang-II internalization upon binding to ACE than to AT_1_ (Bmax in CHO-AT1: 69.6 ± 10.3%; Bmax in CHO-ACE: 79.6 ± 5.7%, n = 3, p<0.05), (**Fig 1A**). In order to check if this observation was not due to a matter of a difference on the expression level of AT_1_ and ACE, we performed western blotting and observed that the total expression levels of ACE and AT_1_ was the same (CHO-AT_1_: 0.46 ± 0.045 *vs* CHO-ACE: 0.42 ± 0.07), (**Fig 1B)**. Similarly, the amount of internalized Ang-II-FITC on CHO-ACE was significantly higher than that on CHO-AT_1_ (CHO-ACE: 69.2 ± 9.3 a.u. *vs* CHO-AT_1_: 22.6 ± 1.3 a.u., n = 3, p<0.01), (**Fig 1C)**. Cell membranes of both cells were counterstained with Wheat Germ Agglutinin (WGA). The purpose of using WGA as a probe is that it is a member of the lectin family that binds to N-acetyl-D-glucosamine and sialic acid residues found on the surface of cell membranes. It has been used extensively to stain surface of membranes [26] better than the usage of phalloindin toxins, for example, which in turn label the actin cytoskeleton, providing an indirect labeling of the membrane and not this component itself. A WGA staining of unstimulated cells is shown in order to exclude any possible membrane reorganization due to Ang-II (**Fig 1D).**

Additionally, we observed that after Ang-II stimulation, ACE itself is also routed to the nucleus (**Fig 1E**). Nuclear and non-nuclear protein fraction, before and after Ang-II stimulation, confirmed the translocation of ACE from the cell membrane into the nuclear compartment (Nuclear fraction: Control = 0.1 ± 0.001 a.u., 5 min = 0.99 ± 0.09 a.u., 15 min = 1.08 ± 0.07 a.u.; Non-nuclear fraction: Control = 1.10 ± 0.01 a.u., 5 min = 0.94 ± 0.03 a.u., 15 min = 0.49 ± 0.04 a.u.; n = 3, p<0.01), (Fig **1F** and **1G**). Together, these results show that Ang-II induces internalization of ACE, and a subpopulation of these complexes translocates to the nucleus.

### Phospholipase-C mediates the Ca^2+^ signals induced by ACE activation

**Fig 2:**
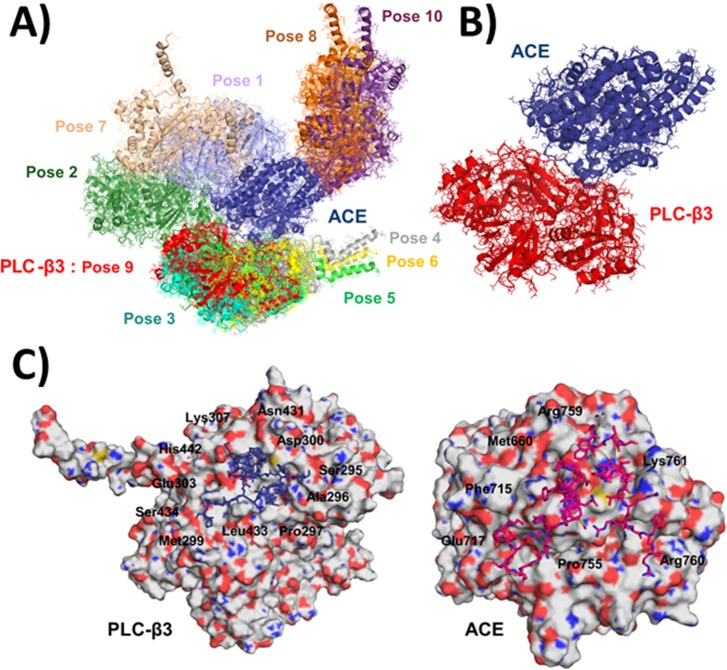
Molecular interaction between ACE and PLC by computational analysis in silico. (A) Representation of the ten top ranked docking poses for ACE (blue) with PLCβ3 protein superimposed (the color of the PLC protein pose correspond to the colors of the labels). (B) Structure of the complex between ACE (blue) and PLC-β3 (Pose 9, red). The binding energy for the best docking pose of ACE and PLC-β3, Pose 9, is -194.06 kcal/mol. C) Amino acid residues located at the interface between the best docking pose of PLC-β3 (left panel) and ACE (right panel), Pose 9, explored through docking protocols.

The binding of Ang-II to ACE is known to cause intracellular Ca^2+^ increase, through InsP_3_ formation [7]. We then investigated whether the membrane-associated phospholipase C (PLC) plays any role in the InsP_3_-mediated Ca^2+^ signals induced by ACE activation. Our *in silico* modeling studies of ACE [27] and PLC Beta 3 (β3) isoform [28] structures, suggested that there is a direct interaction between these molecules, which is independent of G-protein (**Fig 2A–2D**). The ten top ranked docking poses obtained with ZDOCK are presented in **Fig 2A**. The interaction energy obtained for the best docking pose of PLCβ3, Pose 9 (**Fig 2B)**, possess an energy value of -194.06 kcal/mol, within the Universal classical force field. **Fig 2C** shows the amino acid residues located at the interface between the best docking pose of PLCβ3 (left panel) and ACE (right panel), Pose 9, explored through docking protocols.

**Fig 3:**
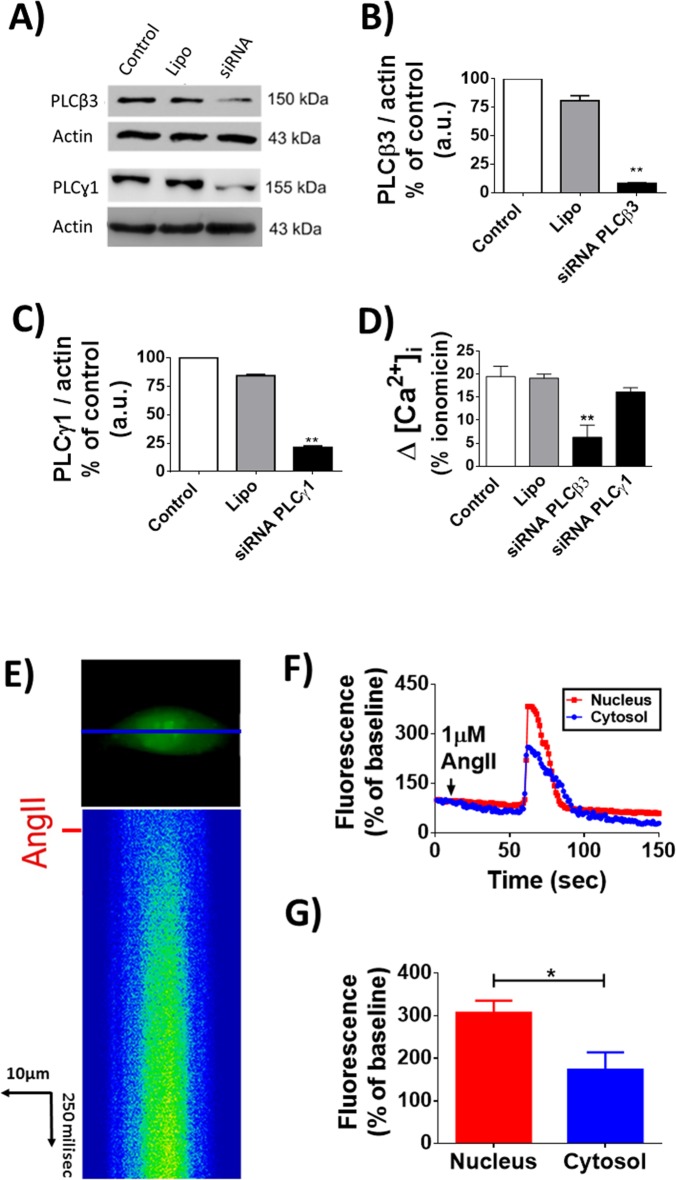
Effect of Ang-II on intracellular Ca2+ signaling in CHO-ACE cells. (A) Western immunoblotting to confirm the silencing of PLC isoforms. Densitometric analysis are shown on (B) for PLCβ3 and (C) for PLCγ1. (D) Quantitative representation of intracellular [Ca^2+^] in CHO-ACE cells transfected with siRNA-PLCβ3 and PLCγ1 using Lipofectamine and stimulated with Ang-II (1μM). (E) Line scanning of Ca^2+^ signal in CHO-ACE. Ang-II promotes Ca^2+^ increase with greater intensity in the nuclear region. (F) Time course of Ca^2+^ signaling in the nucleus (red traces) and cytosol (blue traces). (G) Quantification of fluorescence intensity signal in the nucleus and cytosol. Values are mean ± S.E.M., (* p<0.01), 45 cells, n = 3 individual experiments.

The importance of this interaction between PLC and ACE was experimentally validated by silencing of specific PLC isoforms followed by evaluation of Ang-II triggered Ca^2+^ signals in CHO-ACE cells. The effectiveness of silencing PLCβ3 or the PLC gamma (γ) isoforms was confirmed by western blotting (PLCβ3: Control = 100% *vs* siRNA PLCβ3 = 12 ± 0.15%; PLCγ: Control = 100% *vs* siRNA PLCγ = 24.5 ± 0.36%, n = 5, p<0.01 for each condition), (**Fig 3A–3C)**. Silencing of PLCβ3 but not PLCγ in CHO-ACE decreased Ca^2+^ signaling induced by Ang-II (Control: 19.52 ± 2.2%, Lipo: 19.15 ± 0.85%, siRNA PLCβ3: 16.6 ± 1.9% and siRNA PLCγ: 16.57 ± 0.12% n = 3, p<0.01), (**Fig 3D)**. Additionally, Ca^2+^ signals induced by Ang-II/ACE activation initiate and predominate in the nuclear compartment (Nucleus: 166.6 ± 43.2% *vs* Cytosol: 49.5 ± 15.8%, n = 3, p<0.05), **(Fig 3E–3G)**. Together, these results indicate that upon Ang-II stimulation, ACE interacts with PLC to trigger an intracellular Ca^2+^ increase predominantly in the nucleus of CHO cells.

### Clathrin regulates endocytosis of ACE

**Fig 4:**
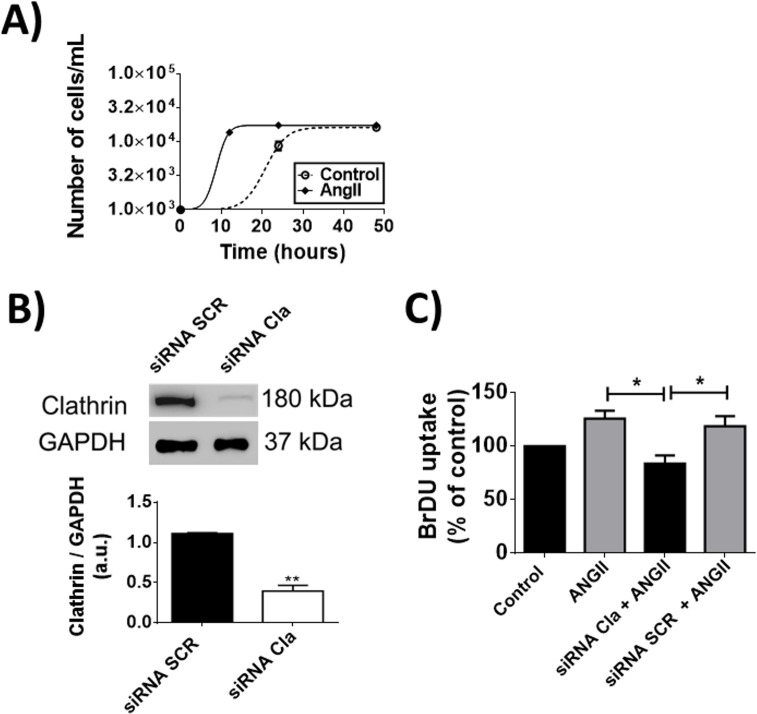
Cell proliferation induced by Ang-II/ACE involves clathrin- mediated internalization process. (A) Cell growth assay of CHO-ACE cells 12, 24 and 48 hours after stimulation with Ang-II (1μM), triplicate in 3 individual experiments. (B) Western blot to confirm the silencing of clathrin (upper panel) and densitometry analysis (bottom panel). Mean ± S.E.M., n = 5 (* p<0.05). (C) BrDU incorporation is decreased in CHO-ACE cells transfected with siRNA-Cla (Clathrin) and stimulated with Ang-II (1μM). Mean ± S.E.M., n = 6 (* p<0.05).

Since our previous demonstrated that nucleoplasmic Ca^2+^ regulates cell proliferation [12, 29, 30] and the activation of ACE by Ang-II caused preferential nuclear Ca^2+^ increase, we investigated the role of ACE in cell growth. Ang-II stimulated CHO-ACE cells to proliferate faster compared to control non-stimulated group (CHO-ACE cells reached 1.1 ± 0.28 x 10^4^ cells *vs* 1.5 x 10^3^ ± 0.25 cells from the control group, after 15 hours of incubation in the presence of Ang-II, n = 4, p<0.05), (**Fig 4A)**. Next, we investigated whether the nuclear translocation of ACE is necessary for the proliferative response. For that, we performed silencing of clathrin (Cla), which is involved in classic receptor endocytosis [31]. Our result showed an efficient silencing of Cla in CHO-ACE cells (siRNA SCR: 1.39 ± 0.01 *vs* siRNA Cla: 0.46 ± 0.025), (**Fig 4B)**. BrDU assay showed that silencing of Cla inhibited cell proliferation stimulated by Ang-II on CHO-ACE cells (Data expressed as percentage of the control group. Ang-II: 25 ± 4.34%, siRNA Cla + Ang-II: 0 ± 5.4%, siRNA Scr + Ang-II: 20 ± 6.56%, n = 3, p<0.05), **(Fig 4C)**. These results demonstrate that clathrin-mediated endocytosis is necessary to promote cell proliferation induced by Ang-II.

### Ang-II/ACE regulates melanoma cell proliferation and migration

**Fig 5:**
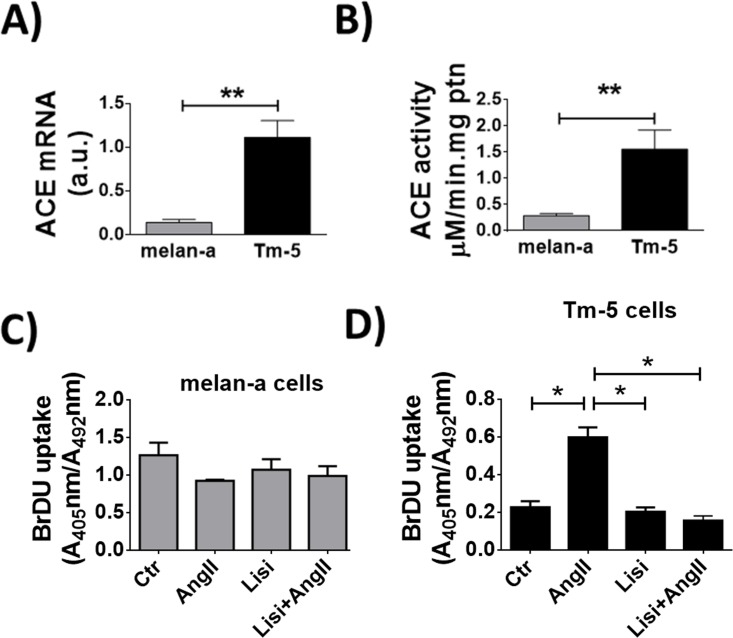
Ang-II stimulates proliferation in melanoma cells. (A) Real-Time PCR analysis for expression of ACE in melan-a and TM-5 cells. (B) ACE activity measured by cleavage of Abz-FRK(Dnp)P-OH in melan-a and TM-5 cells. For A and B, mean ± S.E.M, n = 6 (**p<0.01). (C-D) BrDU uptake assay 24 hours after stimulation with Ang-II (1μM) in the presence of lisinopril (1μM), showing inhibition of cell proliferation in TM-5 cells (D) but not in melan-a cells (C). Mean ± S.E.M., n = 6. (*p<0.05).

To further investigate the role of ACE on cell proliferation, we used the murine melanoma cells (TM-5), since TM-5 cells endogenously express ACE, but do not express AT_1_ and AT_2_ receptors [32]. ACE expression level is higher in TM-5 cells than in its control melanocyte counterpart, Melan-a cells (TM-5 cells: 1.1 ± 0.1 a.u. *vs* Melan-a cells: 0.14 ± 0.05 a.u., n = 5, p<0.01), (**Fig 5A**). As expected, ACE activity is also more pronounced in TM-5 compared to Melan-a (0.42 ± 0.1 in melan-a *vs* 1.80 ± 0.42 in TM-5, n = 3, p<0.05), (**Fig 5B**). No proliferation was observed in response to Ang-II stimulation in Melan-a cells (Control: 1.3± 0.2 Ang-II: 0.9 ± 0.1, Lisi: 1.2 ± 0.3, Lisi + Ang-II: 1 ± 0.2, n = 3, p = ns), (**Fig 5C**). Conversely, BrDU incorporation was increased in TM-5 upon Ang-II, an effect that was prevented by pre-treatment with ACE inhibitor, Lisinopril (Control: 0.2± 0.1, Ang-II: 0.6 ± 0.1, Lisi: 0.2 ± 0.1, Lisi + Ang-II: 0.19± 0.1, n = 3, p = <0.01), (**Fig 5D**).

**Fig 6:**
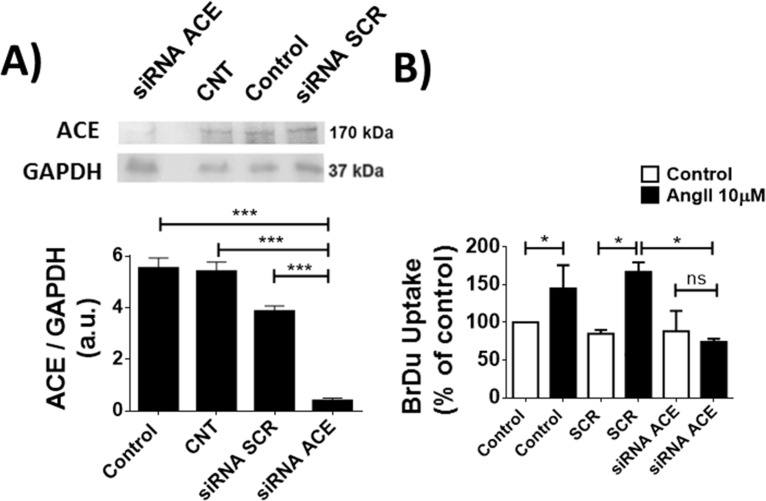
ACE silencing inhibits the proliferative effect of Ang-II in melanoma cells. (A) Western bot (upper panel) to confirm the silencing of ACE and densitometry analysis (bottom panel). Mean ± S.E.M, n = 8. (***p<0.01 compared to respective columns). (B) BrDU uptake in Tm5 cells silenced for ACE and stimulated for 24 hours with Ang-II (1μM), showing a decrease in BrDU incorporation in the absence of ACE. Mean ± S.E.M., n = 12 (*p<0.05; ns = non-significant).

To address the potential direct involvement of ACE in TM-5 cell proliferation, ACE expression was silenced in this cell type (Control: 5.93 ± 0.2%, CNT: 5.78 ± 0.16%, siRNA SCR: 4 ± 0.1% and siRNA ACE: 0.3 ± 0.01%, n = 3, p<0.01), (**Fig 6A)**. In cells with reduced levels of ACE, Ang-II was unable to induce TM-5 cell proliferation (Control: 147 ± 23%, siRNA SCR: 172 ± 9% and siRNA ACE: 0 ± 2%, n = 4, p<0.05), (**Fig 6B**), indicating that ACE activation is involved in melanoma proliferative response triggered by Ang-II.

**Fig 7:**
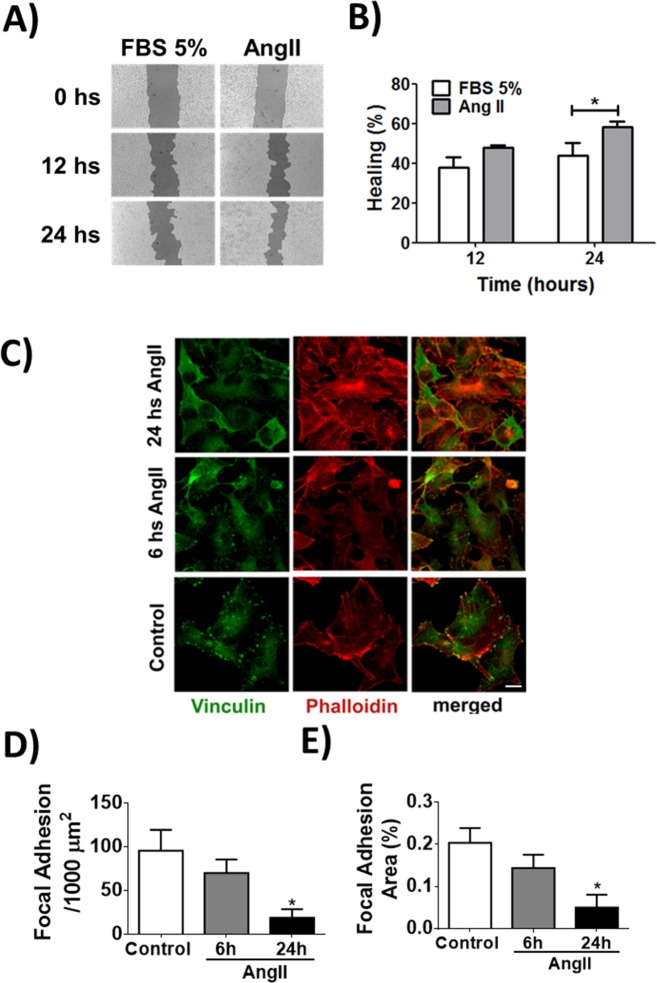
Ang-II promotes cellular migration and reduces focal adhesion formation in melanoma cells. (A-B) Wound healing assay using TM-5 cells stimulated with Ang-II (1μM). C) Representative confocal images of TM-5 cells double-labeled with vinculin (green) and phalloidin (red). Scale bar = 10 μm. (D-E) Quantification of the focal adhesion formation. Mean ± S.E.M., n = 6 (* p<0.05).

Melanoma is the most aggressive form of skin cancer [33]. When it has spread (metastatic melanoma), the prognosis is very poor. Indeed, this type of skin cancer has a great capacity of migration [33]. To investigate whether Ang-II induces migration of melanoma cells, we performed a scratch assay in the presence or absence of Ang-II. Ang-II stimulateed a faster healing process (Results expressed as the percentage of healing. FBS 12h: 37.7 ± 3.1%, Ang-II 12h: 47.9 ± 4.4%, FBS 24h: 44 ± 5.1%, Ang-II 24h: 58.2 ± 7.2%), (**Fig 7A and 7B**). Some recent findings showed that the filamentous (F)-actin-binding protein vinculin is required for cell polarization and migration [34], having a key role on the formation of focal adhesion points. In order to check if the increased healing observed during stimulation with Ang-II is due to alterations on the focal adhesion points, we performed immunofluorescence for vinculin. Under stimulation with Ang-II, TM-5 cells showed a decreased focal adhesion formation/1000μm^2^ (Control: 95.5 ± 23, Ang-II 6h: 69.9 ± 15.4, Ang-II 24h: 18.7 ± 9.8, n = 5, p<0.05), (**Fig 7C–7E**). Taken together, these data suggest that the reduction of focal adhesion observed in Ang-II stimulated melanocytes might contribute to the detachment and migration of melanoma cells.

## Discussion

The major form of somatic ACE involves two very similar protein domains (N- and C-domains) [35, 36], with the C-domain of ACE responsible for Ang-II formation [37, 38], the principal final product of the renin-angiotensin system. However, ACE is a relatively nonspecific peptidase that is capable of cleaving a wide range of substrates. Because of this, ACE and its peptide products affect many physiologic processes, involving blood pressure regulation, handling of electrolytes in the kidneys, hematopoiesis, reproduction, immune response among others [39]. Recently, evidence has been provided that ACE has kinase activity [4, 5] and also functions as a membrane receptor for Ang-II [7, 9]. Indeed, crystal structure of ACE c-domain in complex with Ang-II revealed detailed molecular interaction site between ACE and the peptide [9]. Once bound to ACE, Ang-II was shown to cause an InsP_3_-dependent intracellular Ca^2+^ increase [7]. In the current work, we showed that through ACE, Ang-II increases Ca^2+^ signals preferentially in the nucleus and modulates cellular functions such as proliferation and migration. We also demonstrated that ACE and Ang-II complex internalizes in a clathrin-dependent way.

Upon activation by ligand stimulation, several membrane receptors are internalized through the clathrin-coated endocytic pathway, suggesting an intranuclear signaling of both ligands and receptors [40]. For AT_1_ receptor, its internalization is initiated by G protein-coupled receptors (GPCRs) kinases that promote phosphorylation of aminoacid residues within the cytoplasmic tail of the receptor [41]. After that, the phosphorylated receptor can interact with arrestins (β- arrestins-1and-2), which in turn, promotes entry of the receptor into clathrin-coated pits [42]. Although we showed Cla took part on the ACE internalization, we have no evidences whether ACE interacts with β -arrestin in order to entry on clathrin-coated pits, as it has already been demonstrated for AT_1_ and other GPCRs [41, 43, 44, 45].

In the endosome-mediated nuclear translocation, the internalized endosome is usually directed by the nuclear transporter importin proteins, which recognize the nuclear localization signal of the proteins and direct them to the nucleus through the nuclear pore complex [46, 47]. However, the nuclear localization signal is not predicted in the entire ACE sequence [48], therefore nuclear import of ACE may require other mechanisms. Indeed, several receptors tyrosine kinases (RTKs), that lack the nuclear localization signal, have also been reported to localize in the nucleus [48]. In this aspect, we have previously described that the nuclear accumulation of the hepatocyte growth factor (HGF) receptor requires Gab1 participation [13]. Gab1 is an adaptor protein that contains a nuclear localization sequence and by binding to the target protein regulates its active import to the nucleus [49]. It is possible that a similar mechanism mediates ACE nuclear translocation. Additional questions include whether Ang-II remains bound to nuclear ACE and whether the nuclear localization of ACE may take place only at specific stages of the cell cycle. While more studies need to be done to characterize the nuclear translocation of ACE/Ang-II complex, we are now demonstrating that this internalization process is essential to cause a preferential nuclear Ca^2+^ increase.

Intracellular Ca^2+^ signals mediated by InsP_3_ rely on activation of PLC that causes hydrolysis of phosphatidylinositol bisphosphate (PIP_2_) [50]. This activation typically depends on heterotrimeric G protein subunits, but can also be triggered by protein tyrosine kinases, small G proteins, and phospholipids [51]. We now show that ACE may directly interact with PLC, and that in CHO cells, the PLCβ3 isoform, but not the PLCγ1, regulates the Ca^2+^ signals triggered by Ang-II/ACE pathway. One can thus speculate that the subpopulation of activated ACE that translocates to the nucleus might locally activate PLCβ3 to generate nuclear Ca^2+^ signals upon Ang-II stimulation. This is supported by data from other cell systems, which show expression of PLC family members not only at the plasma membrane, but also preferentially in the inner nuclear compartment [52]. Furthermore, a link between nuclear inositol lipid cycle and nuclear Ca^2+^ signal is well established, and the activation of this pathway has been shown to act independently from that at the plasma membrane [13, 53–58]. Moreover, it is known that mitogens such as insulin [53] and hepatocyte growth factor, once bound to their respective receptors [13] can lead to hydrolysis of the nuclear pool of PIP_2_, through a PLC-dependent mechanism, leading to a nuclear Ca^2+^ increase [50]. It is conceivable that a similar phenomenon occurs with ACE/Ang-II. However, since the selectively silencing of PLCβ3 did not completely abolish intracellular signals triggered by Ang-II and ACE interaction, we cannot exclude a partial contribution of other signaling molecules as well. Indeed, Guimarães and Alvarenga, 2011 [7] showed that either 2-APB (InsP_3_R antagonist) or Nifedipine (L-type voltage-gated Ca^2+^ channel blocker) were able to partially block ACE-evoked Ca^2+^ signaling in CHO-ACE cells, suggesting that, besides mobilizing intracellular Ca^2+^ stores, Ang-II binding to ACE also affects the opening of the voltage-gated Ca^2+^ channels. Therefore, the partial blockage of Ca^2+^ signaling due PLCβ signaling dumight be explained due the different Ca^2+^ pathways that are activated by Ang-II binding to ACE. Another possibility is that ACE/Ang-II links to a specific isoform of the InsP_3_R to trigger nuclear Ca^2+^ release. For instance, it is known that three InsP_3_ receptor isoforms exist and they have distinct sensitivity to InsP_3_ [50] and subcellular localizations, which would enable InsP_3_-mediated Ca^2+^ signals to occur preferentially in the nucleus, compared to the cytosol, after ACE activation [50].

Ca^2+^ signals within the nucleus are particularly important in cancer cell progression [50, 59, 60]. Buffering nuclear Ca^2+^ arrests adenocarcinoma cells in the early phase of mitosis [61], and sensitizes head and neck cancer cells to radiotherapy [59]. Therefore, the effect of Ang-II/ACE on nuclear Ca^2+^ signaling might explain the observed Ang-II’s action as a mitogen, in the melanoma cell line (TM-5), a murine cell type that endogenously expresses ACE, but lack Ang-II type 1 or type 2 receptors. We further show that ACE is involved in TM-5 cell migration, another aspect of melanoma carcinogenesis. Melanoma is a common cancer in the Western world with an increasing incidence highly due to sun exposure [62]. Transformation of melanocytes into melanoma encompasses a complex interplay of both endogenous and exogenous factors and it is known that its metastasis pattern can occur during either an earlier or a later phase, being guided by genetic or phenotypic drivers [63, 64]. Although Ang-II has been reported to regulate growth, adhesion, invasion and cell migration in certain cancer cells [65], this is the first report of Ang-II-induced melanocytes proliferation and migration mediated by binding to ACE.

It is well-accepted that either ACE inhibitors or AT1 blockers are the gold standard drugs in order to manage hypertension, due their survival benefits provided on patients with heart failure, high cardiac risk profile and also proteiunuric chronic kidney disease [66]. However, a combination therapy with ACE inhibitors and Ang-II receptor blockers has been extensively explored since the monotherapy has been shown efficient in only a quite limited number of hypertensive patients [67]. Another evidence that supports the usage of a combined therapy is the fact that monotherapy with ACE inhibitor increases the concentration of circulating Ang-I and this can partially mitigate inhibition of ACE, what turns out to restore the concentration of active Ang-II towards pretreatment levels [68, 69]. In addition to that, it is known that other enzymes distinct from ACE, and therefore not blocked by ACE inhibitors, can form Ang-II [70]. Indeed, patients with mild to moderate hypertension demonstrated a more prominent decrease in diastolic blood pressure when a combination therapy was used [71, 72]. However, the long-term effects of these combination therapies on blood pressure have still been questioned since they showed no benefits in terms of the composite of cardiovascular death, myocardial infarction, stroke and hospitalization for heart failure. In fact, it caused more symptoms attributable to hypotension, increased decline in renal function and need for dialysis compared to ACE inhibitor monotherapy [73].

There are several clinical evidences showing the chemopreventive effects of ACE-blocking in cancer [74]. The first evidence for the antitumor effects of ACE blockers was demonstrated in 1998 [75] in which the relative risk of fatal, incident and female-specific cancers was lower in women on ACE inhibitors [75]. In human squamous skin cancer cells, it was observed a prominent inhibitory effect on tumor growth and angiogenesis mediated by perindopril [76]. A similar finding was observed in a cohort study performed among a high-risk group of veterans using ACE inhibitors, showing a lower incidence of keratinocyte cancer when compared to nonusers. [77]. Specifically for cutaneous melanoma, captopril presented an antitumor activity in human melanoma xenograft model [78]. Additionally, it is already known that AT1 receptor plays an important role in angiogenesis and growth of tumor cell [79]. Administration of the AT1 blocker, TCV-116, significantly decreased melanoma tumor volume in mice [80]. However, more clinical studies are still needed in order to justify the usage of ACE inhibitors or AT1 blockers for treating melanoma and other malignancies.

Taken together, our findings here suggest a novel function of ACE in the pathology of melanoma and open new paths to further studies, where ACE, as a receptor, might function as a possible therapeutic target aiming to avoid the progression of the disease.
